# Resonance Energy Transfer-Based Biosensors for Point-of-Need Diagnosis—Progress and Perspectives

**DOI:** 10.3390/s21020660

**Published:** 2021-01-19

**Authors:** Felix Weihs, Alisha Anderson, Stephen Trowell, Karine Caron

**Affiliations:** 1 CSIRO Health & Biosecurity, Parkville, 343 Royal Parade, Melbourne, VIC 3030, Australia; felix.weihs@csiro.au; 2 CSIRO Health & Biosecurity, Black Mountain, Canberra, ACT 2600, Australia; alisha.anderson@csiro.au; 3 PPB Technology Pty Ltd., Centre for Entrepreneurial Agri-Technology, Australian National University, Canberra, ACT 2601, Australia; sct.ppbtech@icloud.com

**Keywords:** FRET, BRET, CRET, point-of-care, on-site, on-the-spot, microfluidics, PADs, time-resolved FRET

## Abstract

The demand for point-of-need (PON) diagnostics for clinical and other applications is continuing to grow. Much of this demand is currently serviced by biosensors, which combine a bioanalytical sensing element with a transducing device that reports results to the user. Ideally, such devices are easy to use and do not require special skills of the end user. Application-dependent, PON devices may need to be capable of measuring low levels of analytes very rapidly, and it is often helpful if they are also portable. To date, only two transduction modalities, colorimetric lateral flow immunoassays (LFIs) and electrochemical assays, fully meet these requirements and have been widely adopted at the point-of-need. These modalities are either non-quantitative (LFIs) or highly analyte-specific (electrochemical glucose meters), therefore requiring considerable modification if they are to be co-opted for measuring other biomarkers. Förster Resonance Energy Transfer (RET)-based biosensors incorporate a quantitative and highly versatile transduction modality that has been extensively used in biomedical research laboratories. RET-biosensors have not yet been applied at the point-of-need despite its advantages over other established techniques. In this review, we explore and discuss recent developments in the translation of RET-biosensors for PON diagnoses, including their potential benefits and drawbacks.

## 1. Introduction

Over the last 50 years, there has been a substantial trend to developing point-of-need (PON) diagnostic testing, also known as “on-the-spot” or “point-of-care”, putting rapid testing into the hands of first responders, processors and consumers. The development of such diagnostics is seen across healthcare [1,2], food and beverage [3,4], and industrial quality control applications [5,6]. Ideally, a PON test delivers accurate and repeatable results in short time periods, with minimal sample preparation, minimal user expertise and minimal resource requirements. PON testing can be enabled by the miniaturization of a range of technologies such as ultrasound [7], MRI [8] and spectroscopy [9]. However, imaging and spectroscopic technologies tend to be limited to anatomically apparent pathologies and require significant on-site analysis and end-user expertise to interpret results. Biosensors that combine a biological recognition element with a transduction modality for the detection of a range of analytes are ideally placed to meet the requirements for accurate and repeatable PON diagnostics without deep analytical expertise on the part of the end-user. The biological recognition elements selected are proteins or nucleic acids that have been honed by natural selection to be highly sensitive and specific for their target analytes. The range of such elements includes enzymes, antibodies, receptor proteins, DNA and RNA. Biological recognition elements can be coupled to a mechanical, electrical or optical transduction mechanism, to enable rapid signal readouts, and many different biosensor transduction mechanisms have been used in research and diagnostic laboratories. For example, fluorescence [10], Surface Plasmon Resonance (SPR) [11] and Surface Enhanced Raman Spectroscopy (SERS) [12] have recently attracted significant interest in research for PON biosensing. However, only two transduction mechanisms have been widely adopted at the PON, namely lateral flow immunoassay (LFI) and electrochemical (EC) readouts.

LFI is widely used in many fields, including medicine [13], veterinary [14], food [4], agricultural [15] and industrial process control [16]. As an example, the most commonly used LFI is the pregnancy test, which is available on many pharmacy shelves. The principle of LFIs is based on the movement of a liquid sample via capillary action through a paper-based strip containing antibodies with which analytes of interest interact to produce a colorimetric readout [17]. While LFIs are simple, cheap and portable, the results obtained are qualitative or at best semi-quantitative [18], limiting them to use as a screening tool. Additionally, many LFIs are not sensitive enough to measure trace amounts of analytes in complex samples [19], and they are sometimes unreliable due to the limited control of sample volumes and user error [20,21].

Electrochemical transduction, as the name suggests, utilizes outputs of a voltage or current when biological-recognition elements immobilized on an electrode interact with the target analyte. The best-known application of electrochemical biosensors is for the quantification of glucose for diabetes management [22].

Electrochemical biosensors offer the advantage of simplicity, low cost and reliable quantitative detection even with complex sample matrices. However, they tend to be challenged when detecting trace amounts of analytes and suffer from biosensor drift and surface effects on the electrodes after repeated exposure to biological or chemical matrices, further reducing the sensitivity [23,24]. Very recently, as a means to respond to the worldwide healthcare crisis, rapid detection of SARS-CoV-2 has been demonstrated [10]. With the aim of moving rapid tests from laboratory settings to the PON, CRISPR-based technologies are now being adapted to give either an electrochemical readout [25] or to be carried out on a paper strip [10] similar to that of LFIs.

In this study, we investigated whether there are opportunities for emerging transduction modalities to bring new diagnostic tests to the point-of-care. Specifically, we focused on a class of luminometry, namely Förster Resonance Energy Transfer (RET), that was elaborately developed for research and laboratory use. We explored to what extent, and under what conditions, RET might open up new opportunities in biosensing at the point-of-need.

## 2. Förster Resonance Energy Transfer Sensing Principle

RET is a distance- and orientation-dependent non-radiative energy transfer from an energy donor to an acceptor fluorophore or quencher [26]. Resonance Energy Transfer usually occurs at distances of 1–10 nm, which can vary dependent on the combination of donor and acceptor and the relative orientation of their transition dipole moments [27,28,29]. Due to its extreme sensitivity to spatial changes on a nanometer scale, RET has been extensively used as the transduction modality in lab-based biosensors for the analysis of a wide range of analyte types, such as kinase [30] and protease activity [31,32,33], G-protein-coupled receptors [34,35], antibodies [36], small molecules [37,38] and protein–protein interaction [39,40,41].

**Figure 1:**
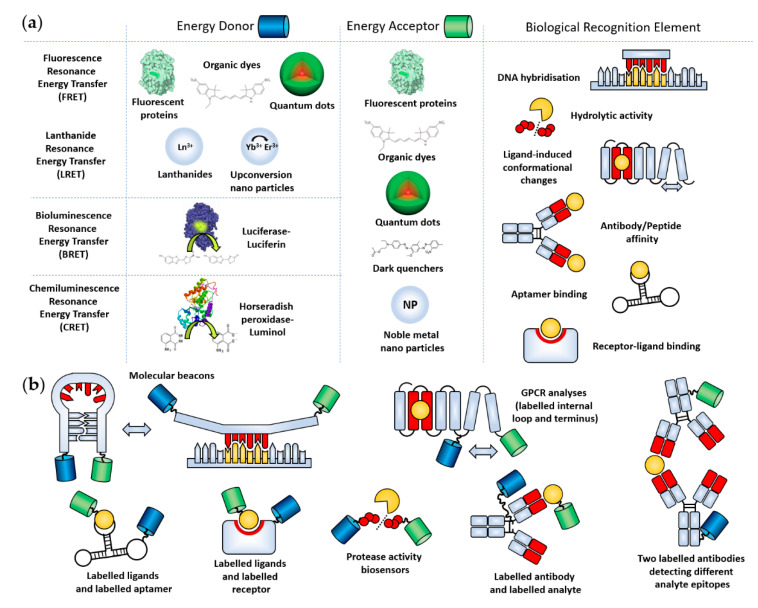
(**a**) Overview of different variations on the Resonance Energy Transfer principle, together with a range of different types of biological recognition element. Red domains indicate recognition elements, and orange domains indicate targeted analytes. (**b**) Examples of different ways that energy donors and acceptors can be combined with biological recognition elements. All illustrations are simplified and not shown to scale. Images were taken from the following sources: The “Fluorescent proteins” image illustrates the Green Fluorescent Protein (doi:10.2210/rcsb_pdb/mom_2003_6). The “Organic dyes” structure shows cyanine. The quantum dot image was taken from Reference [49]. The “Luciferase–Luciferin” image is composed of the Firefly luciferase (doi:10.2210/rcsb_pdb/mom_2006_6) and D-Luciferin as its substrate. The structure of horseradish peroxidase was taken from the Protein Data Bank (1W4E, doi:10.2210/pdb1W4W/pdb). The dark quencher is the black hole quencher BHQ1 from atdbio (https://www.atdbio.com/content/35/FRET-fluorescence-quenchers).

Biosensors have been realized through the incorporation of RET components into biological recognition elements in such a way that the presence of an analyte affects the spatial relation between the donor and acceptor molecules (Figure 1). The versatility of this approach facilitates theoretically infinite options for sensing applications. In contrast, glucose meters rely on a specific enzymatic reaction causing the oxidation of glucose [42]. Such a mechanism cannot be readily translated for the detection of other analytes.

We can identify three different classes of RET according to the identity of the incorporated energy donor: (1) Fluorescence Resonance Energy Transfer (FRET), (2) Bioluminescence Resonance Energy Transfer (BRET) and Chemiluminescence Resonance Energy Transfer (CRET) (Figure 1a). FRET is, arguably, the most frequently applied RET technique in lab-based testing, where energy transfer is initiated through the excitation of a fluorescent molecule, such as a fluorescent protein, organic dye, quantum dot or rare earth element, using an external illumination source. Unlike FRET, BRET and CRET employ endogenous “light” sources by the incorporation of enzymes or chemical catalysts as the energy donor. They require suitable chemical substrates to generate luminescence. Formally speaking, BRET is a subclass of CRET but will be discussed separately because it has several distinct features. The selection of energy donor has profound implications for the technical requirements testing outside the research laboratory, by determining how the optical response of the RET biosensors is initiated. External illumination can generate significant noise due to light scattering, autofluorescence, and bleed through and crosstalk of the exciting radiation. BRET/CRET can therefore deliver reduced noise levels, particularly in complex samples such as blood [43]. Since noise is often limiting for sensitivity, BRET/CRET is potentially well-suited for PON applications, enabling more reproducible measurements and the use of smaller sample amounts. As no external light source component is required, the design of miniaturized BRET/CRET detection devices is simplified compared to in the use of FRET biosensors.

Luciferases have been applied in biolanalytics for decades, but, until recently, bioluminescence and BRET transduction techniques were not considered for point-of-need applications. Their low optical signal output was particularly challenging for miniaturized applications, where low amounts of biosensors are used. However, considerable progress has been made in recent years in the development of brighter and more stable luciferase-luciferin combinations [44,45] coupled to superior acceptors [46,47] and in combination with sensitive BRET detection devices [48].

Enzyme degradation, substrate instability or substrate inhibition can affect the amount of light produced by the luciferase oxidation reaction. However, the variation of emitted photons can be alleviated by using excess substrate and by using ratiometric measurements, as, to a certain extent, a lower light generation is not accompanied by a change in signal ratio. However, some luciferases exhibit flash-type kinetics, demanding a timed detection system, which can be a drawback compared to using FRET as the signal transduction modality.

## 3. Signal Measurements

RET signals within the context of on-site suitable techniques are usually measured either by sensitized emission or fluorescence lifetime. Sensitized emission is commonly applied for all RET systems and describes the measurement of donor and acceptor signal intensities in relation to each other (RET ratio). Close proximity between donor and acceptor molecules results in higher RET ratios than more distant RET components.

Ratiometric measurements exhibit a dose-independent normalization, as the RET ratios for 10 molecules are theoretically the same as for 100 molecules. This makes RET techniques more robust to interfering effects, compared to fluorometric or luminometric approaches, where only the signal of a single fluorophore/luminophore is measured. RET therefore enables homogeneous assays without the need for washing steps. Another way of achieving internal normalization is to analyze the fluorescence lifetime of the donor (time-resolved FRET). The time between donor excitation and photon emission is prolonged if there is RET to an acceptor molecule. Fluorescence lifetimes are usually in the nano- to micro-second range, requiring sophisticated analytical equipment. Fluorescence lifetime is the method of choice for Lanthanide RET (LRET) systems, where rare earth elements with remarkably long fluorescence lifetimes are incorporated as energy donors [49,50].

Whether it is RET ratio or fluorescence lifetime that is measured, these parameters are usually calibrated against standard concentrations of the target analyte, to generate results that are meaningful to the end-user.

## 4. PON-Suitable RET Applications

In a laboratory environment, RET biosensors are usually deployed in micro well plates and read out in a sophisticated plate-reading instrument. Such instruments tend to be too expensive and cumbersome for PON use. Alternative instruments have been developed that generally feature two simplifications. Firstly, micro titer plates are replaced by a medium, such as microfluidic channels, paper-strips or cartridges, that makes it easier to analyze small sample volumes with high reproducibility. Secondly, a PON-specific device is used to detect and interpret RET signals. Approaches include the use of compact integrated devices, modified microscopes or digital cameras.

It is noteworthy that the overall sensitivity of a biosensing test, either lab-based or at the PON, depends on the sensitivity of the biological element used, the transduction modality and the equipment used. In the case of PON, it is possible that sensitivity or quantification may be reduced as a tradeoff for using more convenient equipment. Consequently, the sensitivity of a test depends on all of the components involved. As an indication of the performance of each type of technology, we have listed approximate assay times and sensitivity levels in the respective figures summarizing the technologies reviewed in this article. 

### 4.1. RET Detection Using Microfluidics in Combination with a Compact Detection Device

Microfluidics offers several advantages, such as low cost, reduced reagent consumption, small sample volumes, increased reaction rates and faster analysis times for portable detection devices [51].

#### 4.1.1. Fluorescence Resonance Energy Transfer-Based Systems

**Figure 2:**
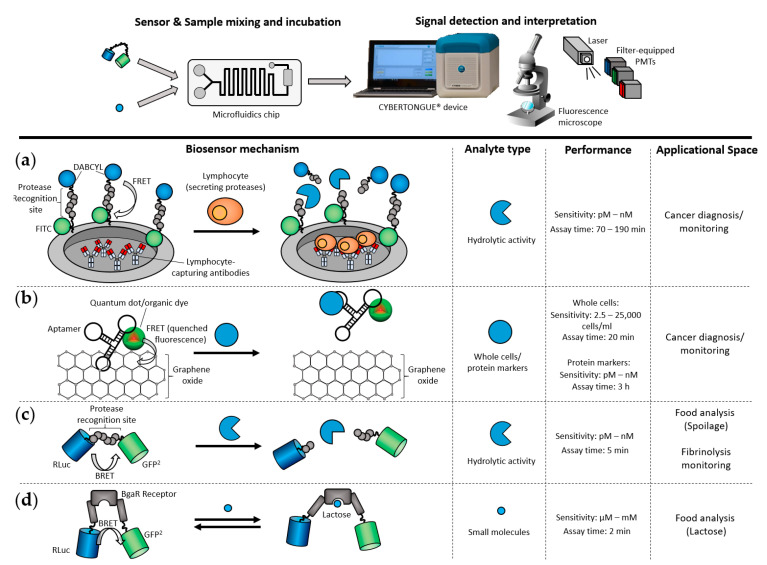
Examples of point-of-need (PON)-suitable applications using compact RET detection devices in combination with microfluidics. In one example, Bioluminescence Resonance Energy Transfer (BRET)-based biosensors are run on a microfluidics chip integrated into a compact device containing micro photon multiplier tubes (µPMTs). In other examples, FRET biosensor signals are recorded by using a fluorescence microscope or laser excitation followed by detection using PMTs. (**a**) Lymphocytes secreting Matrix metalloproteinase-9 (MMP9) are trapped by antibodies in a micro well located on a microfluidic chip. The peptides are labeled with Fluorescein isothiocyanate (FITC) and 4-(dimethylaminoazo)benzene-4-carboxylic acid (DABCYL), and they contain MMP9-specific cleavage sites. These are immobilized close to the micro wells, to detect any MMP9 activity released by the cells [52]. (**b**) Aptamers, labeled with quantum dots (QDs), specific for cancer-related cells or protein markers are attached on a graphene monoxide layer. Binding of the target cells or proteins to their specific aptamer results in a release of the aptamer from graphene oxide, activating the fluorescence signal of the quantum dot/organic dye [53,54]. (**c**) CYBERTONGUE^®^ protease biosensors consist of the Renilla luciferase RLuc8 connected through a peptide linker, containing specific recognition sites for the target protease, to the Green Fluorescent Protein variant GFP^2^. Proteolytic activity exerted on the connecting peptide results in the dissociation of GFP^2^ from RLuc8, leading to a profound change in BRET ratio [48,55,56]. (**d**) The CYBERTONGUE^®^ lactose biosensor consists of a lactose-binding protein tagged with RLuc8 and GFP^2^ that undergoes a conformational change upon binding to lactose [57]. Binding of lactose results in the distancing of the two BRET components, thereby changing the BRET ratio.

The detection of FRET biosensors in PON microfluidic devices has previously relied on using a fluorescence microscope to measure FRET signals. For example, Son et al. developed a microfluidic protease sensing system for monitoring cancer cell function through the release of the cancer-related protease Matrix metalloproteinase-9 (MMP9) [52]. Micro wells containing a hydrogel incorporating a FRET-linked peptide were integrated into a configurable Polydimethylsiloxane (PDMS) microfluidic device and visualized using a fluorescence microscope (Figure 2a). Cancer cells in suspension were trapped by immobilized antibodies in the hydrogel and the activity of MMP9 secreted from the trapped cells was monitored by following the cleavage of the FRET-linked peptides.

Cao et al. [54] developed a high-throughput multi-channel microfluidic chip to detect the interaction of fluorescently labeled aptamers with cancer cells. The surface of the chip was coated with graphene oxide (GO), which non-covalently binds to and quenches the fluorescence of the organic aptamers. Incubation of cancer cells on the chip led to the release of the fluorescent aptamers from the GO coating and recovery of the previously quenched fluorescence (Figure 2b). A single wavelength was used to measure the quantity of released aptamers.

A more compact and miniaturized sensing device for FRET signals used a laser to irradiate the microfluidic chip and optical fibers to deliver the emitted photons to photon-multiplier tubes (PMTs) [53]. This was used to detect three cancer markers simultaneously. Multiplexing was achieved by labeling three different aptamers with quantum dots having distinct emission spectra (Figure 2b). These were bound to GO-coated chips as in the single marker system. Signals were acquired by PMTs equipped with optical band-pass filters tailored to the different quantum-dot emission spectra. This system was capable of quantifying cancer markers in nanoliter-sized droplets of serum. The total assay time was 3 h. This might be too time-consuming to be considered as a classic point-of-need test; however, it could proof useful as a cancer diagnosis tool in remote settings if appropriate follow-up treatments are available. FRET quenching, as used here, does not have the advantages of being ratiometric, as only the activated fluorescence of the donor can be measured. On the other hand, the assay follows a “lights on” format, which is inherently more sensitive than “lights off”.

#### 4.1.2. Bioluminescence-Resonance-Energy-Transfer-Based Systems

Replacing FRET with BRET as the transduction modality markedly decreases optical noise and also means that a source of illumination, such as a laser, is no longer required. The BRET^2^ system, comprising a variant of the *Renilla* luciferase, such as RLuc8, coupled to GFP^2^, a large Stokes-shift variant of the Green Fluorescent Protein, is a highly sensitive RET tool. It exhibits an unusually large Förster distance [28,29] that can increase detection sensitivities, particularly when the radius of the biological recognition element is significantly greater than 1 nm. However, BRET^2^ exhibits low and transient bioluminescence [58] that has to be accommodated for.

Our group initially demonstrated a proof of principle by using a BRET^2^ thrombin sensor deployed in a PDMS microfluidic channel, with the BRET signal relayed through a microscope objective to two filter-equipped PMTs [59]. Subsequently, the microscope was dispensed with and ratiometric BRET^2^ were made in micro liter volumes, using two filter-equipped PMTs combined with fiber optics, in close contact with the microfluidic device [60]. Although this device achieved the measurement of thrombin protease activities in buffer [60] and maltose detection in beer samples [61], it still lacked true point-of-need capability, as it was bulky and did not support on-chip incubation.

**Figure 3:**
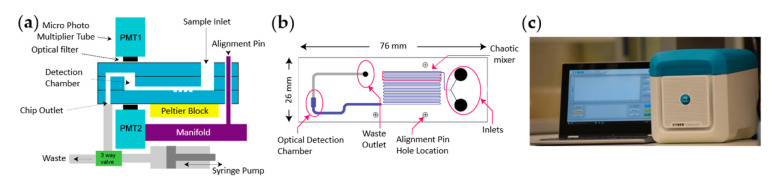
Overview of the Cybertongue^®^ BRET analysis device: (**a**) functional schematic of the measurement device, (**b**) schematic design of a microfluidic chip used for protease assays and (**c**) image of compact microfluidics device with closed lid. Figure was taken from Weihs et al. [48], with permission.

In order to shrink the device’s footprint, micro photon multiplier tubes, instead of large and energy-intensive valve-based PMTs, were placed directly above and below the sample detection chamber. This was implemented within a controlled microenvironment enabled by a thermoelectric block bringing the concept of a compact table top device, termed the Cybertongue^®^ device, to fruition [48] (Figure 3a). The Cybertongue^®^ device is a microfluidics-based platform that can run a variety of homogeneous sensing applications tailored to different analyte types with assay times of 1–10 min. The device combines many of the advantages of the aforementioned examples, such as small sample volumes, ratiometric RET signal measurements, a miniaturized device and rapid analysis times.

The microfluidic channels are etched into a reusable glass chip (Figure 3b) that can be used repeatedly in an on-site setting, benefitting from the fact that glass is relatively inert to a variety of chemicals [62]. Chips with different microfluidic architectures can be inserted into the device, according to the type of assay. Options include a chaotic mixer, followed by serpentine channels, to improve fluid mixing on chip, or an incubation chamber for performing analyte–biosensor pre-incubations, if required.

Captured BRET signals are automatically interpreted via a Bluetooth-connected laptop, which further minimizes handling steps, from sample mixing to results. This setup has allowed the fabrication of a compact sensing system weighing 6.5 kg, suitable for on-site testing (Figure 3c).

#### 4.1.3. Chemiluminescence-Resonance-Energy-Transfer-Based Systems

CRET relies on light emission generated by a redox reaction between luminol and hydrogen peroxide in the presence of a suitable catalyst, such as horse radish peroxidase or metal ions. Its advantages are similar to those of BRET, as it does not require an external illumination source, reducing optical noise, and detection devices can be easily miniaturized [63]. Homogeneous CRET-based assays have been described for the detection of several analytes, including ochratoxin A [64] and thrombin [65,66], using aptamer-mediated analyte binding or for the analysis of estradiol levels [67], C-reactive Protein [68] and Neuron-specific enolase [69] using antibody-based approaches.

However, while a range of commercially available on-site tests using chemiluminescence without RET do exist (allergy tests, flu tests, forensics, air pollutants, etc. [70]), the integration of CRET into suitable on-site testing systems has not been reported. One of the factors that may contribute to this lack of progress is that a number of biologically relevant molecules suppress CRET, such as human serum components [67]; biogenic amines; and thiols, amino acids, organic acids, and steroids [71]. The removal of these molecules via microchip electrophoresis prior analysis could resolve detection issues [71], but potentially adds complexity to a potential on-site test.

### 4.2. RET Detection Using Paper-Based Analytical Devices (PADs) in Combination with a Digital Camera

An alternative pathway to PON-compatible RET technologies is based on paper-based analytical devices (PADs). The low cost, portability and accessibility in low-resource settings of PADs has drawn considerable interest in recent years. Paper-based devices eliminate bulky instrumentation, such as plate readers combined with easy-to-carry-out procedures.

Filter or chromatography paper by Whatman are commonly used for paper-based assays. However, depending on the application, other paper selections can be of importance in the construction of paper-based sensing devices, as differences in porosity, hydrophobicity, pore size, thickness, fiber structure or grammage (mass per unit area) can affect performance of the supported biosensor system [72].

#### 4.2.1. Bioluminescence-Resonance-Energy-Transfer-Based Systems

**Figure 4:**
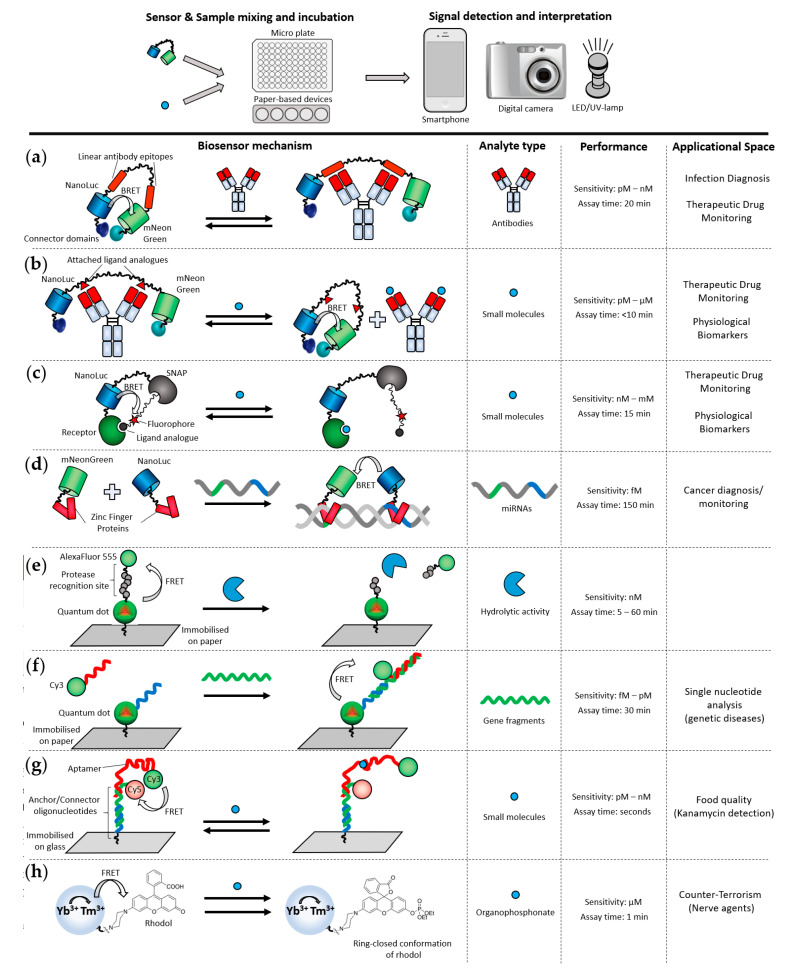
Examples of PON-suitable applications using a digital camera or smartphone in combination with micro plates or paper-based devices. BRET-based biosensors are spotted on paper-based analytical devices (PADs), and signals are recorded with a digital camera or smart phone. FRET biosensors require an additional source of excitation, such as a light-emitting diode (LED) or UV-lamp. (**a**) LUMABS biosensors (LUMinescent AntiBody Sensor) are comprised of the luciferase NanoLuc and the fluorescent protein mNeonGreen connected through a linker containing linear epitopes for antibodies of interest. In the absence of the antibody of interest, NanoLuc and mNeonGreen dimerize through the connector domains [73,74,78]. (**b**) LUMABs were modified by replacing linear epitopes with unnatural amino acids acting as a chemical handle to introduce analogues of analytes of interest. An antibody binding to these analogues is introduced, separating NanoLuc and mNeonGreen. In the presence of the analyte, antibodies preferentially bind to the analyte instead of its analogues incorporated in the LUMAB biosensor [77]. (**c**) LUCIDs (luciferase-based indicators of drugs) are protein fusions comprising NanoLuc, a receptor protein for the drug of interest and the self-labeling enzyme SNAP. A SNAP-functionalized organic dye Cy3 is attached to an analyte analogue, which is covalently incorporated by the SNAP protein. In the presence of the analyte, the receptor preferentially binds the analyte over its SNAP–Cy3-bound analogue [79,80,81]. (**d**) If target miRNAs are present in a sample, miRNA templates are amplified through a rolling circle amplification (not illustrated). Complementary oligonucleotides form double-stranded DNAs that are recognized by fusions of NanoLuc and mNeonGreen with zinc finger proteins that specifically bind to different but nearby sequences [82]. (**e**) Quantum dot (QD)–organic fluorescent dye conjugates joined by a peptide-containing protease-specific recognition site are immobilized on paper. In the absence of proteolytic activity, FRET occurs between the QD and the organic dye, resulting in a yellow/orange emission signal. If the peptide is cleaved due to the proteolytic activity of the protease of interest, the dye diffuses away from the QD, leading to a green emission from the QDs [83]. (**f**) Paper-immobilized quantum dot–oligonucleotides and free Cy3–oligonucleotides contain different DNA segments complementary to the target gene fragment. In a sandwich format, the target gene serves as a hybridization bridge for the QD–oligonucleotide and Cy3–oligonucleotide, which in turn enables FRET between QD and Cy3 [84,85,86,87]; (**g**) A Cy3-labeled kanamycin-specific aptamer partially hybridizes to an anchor/connector oligonucleotide immobilized on glass. The connector oligonucleotide is conjugated to Cy5. Binding of kanamycin spatially separates Cy3 from Cy5 components, leading to a lower FRET efficiency [88]. (**h**) An upconversion nanoparticle (UCNP) consisting of ytterbium (Yb^3+^) and thulium (Tm^3+^) is conjugated to the organic dye rhodol. FRET occurs between the UCNP and rhodol, while organophosphonates perform a nucleophilic attack on rhodol, inactivating it as a FRET acceptor [89].

Antibodies are important biomarkers for the diagnosis and surveillance of fast-evolving infectious diseases and life-threatening allergies. Due to the urgency associated with such medical conditions, PON detection of antibody biomarkers could offer rapid answers and better health outcomes. An example of PON antibody detection was demonstrated for LUMABs (LUMinescent AntiBody Sensor) BRET-based biosensors (Figure 4a) [73,74]. These biosensors incorporate the highly luminescent luciferase NanoLuc [75] that enables BRET signal detection beyond a compact and controlled environment, such as in the Cybertongue^®^ device. To directly quantify antibody in blood plasma, the authors were first able to carry out the homogenous BRET assay in solution, using a smartphone to measure the photon output within 20 min. To further improve the applicability of the test at PON, they then developed and applied the antibody assay on a paper support [74]. This fully integrated paper-based analytical device is composed of vertically assembled functionalized layers that facilitate blood plasma separation. This reduces the handling steps and simply requires the application of a drop of blood on the device. Similar to the corresponding homogenous assay, the results are obtained within 20 min, following photographic analysis with a smartphone. This system enables highly sensitive measurements of three antibodies against HIV, influenza and dengue, in blood, at low nanomolar concentrations (2.8 nM–19.3 nM detection limits). However, the LUMABS design is only applicable to antibodies that recognize linear epitopes, excluding the majority of antibodies that bind to conformational epitopes [76]. LUMABS were further developed to enable the detection of small molecules, such as dinitrophenol or creatinine, by the introduction of non-natural amino acids (pAzF) in place of the linear epitopes [77] (Figure 4b). This strategy can also be used to sense antibodies binding to conformational epitopes, if those are small molecules. However, paper-based detection of pAzF-LUMABS biosensors has not been reported yet.

A different sensor design also utilizing the NanoLuc luciferase, termed Luciferase-based indicators of drugs (LUCIDs), was followed by Johnsson and co-workers for the monitoring of small molecule drugs [79] and later applied for use on paper-based strips, in combination with a digital camera [80,81]. For this purpose, lyophilized biosensors were mounted onto filter paper and liquid samples, such as human serum or whole blood including the luciferase substrate, were added to the paper strip. The reaction between the biosensor and the analyte occurs on paper, and the bright luminescence of NanoLuc is detected by taking a photograph of the paper strip, followed by a software-guided photon analysis. The combination of the lyophilized stabilized biosensor system and the use of low-tech, easily accessible analytical equipment, such as a digital camera, facilitates the translation of such a technology to point-of-need. Following this first LUCIDs PON application, a range of LUCID biosensors were developed for the detection of analytes, such as phenylalanine or glutamate [80] and the clinical drugs methotrexate, theophylline and quinine (Figure 4c) [81]. The detection of such analytes in whole blood at PON is of high interest due to their clinical relevance for monitoring children and pregnant women suffering from phenylketonuria.

MicroRNAs (miRNAs) provide vital information about many diseases and are of great interest in the diagnosis and monitoring of cancer [90]. Analyses of miRNAs usually require a time-consuming amplification step and a PCR instrument. Wu and co-workers recently developed a BRET-based point-of-care suitable technique for the detection of miRNAs [82]. This was achieved by coupling a paper-based isothermal rolling circle DNA amplification with detection by biosensors incorporating NanoLuc and mNeonGreen fused with DNA sequence-specific Zinc Finger Proteins (ZFPs). Human serum can be applied on a paper disc containing lyophilized components required to amplify the target miRNAs through circular single-stranded DNA amplicons. Only the presence of the target miRNAs, acting as primers, enables the amplification of the DNA amplicons. Subsequently, another paper disc, one that contains miRNA complementary oligonucleotides and BRET biosensors, is applied on top of the amplification disc. If miRNAs were present in the sample, the complementary oligonucleotides form double-stranded DNAs with the amplicons that are, in turn, recognized by NanoLuc-ZFP and mNeonGreen-ZFP (Figure 4d). In absence of the miRNAs, both fusions remain dispersed and no BRET can be observed. Signals were detected via a smartphone camera. The speed of point-of-care diagnosis can be less relevant to cancer diagnoses, since these usually do not require quick diagnoses. However, such a system might be of value when it important to monitor biomarkers during treatment visits or to diagnose in remote and resource-limited settings.

#### 4.2.2. Fluorescence-Resonance-Energy-Transfer-Based Systems

A range of FRET biosensors have been developed for paper-based devices and analyzed by using a digital camera. For instance, Petrayayeva et al. developed a paper-based assay to monitor protease activities at the PON or in low-resources settings [83]. The biosensor consisted of on-paper spots of quantum dots (QDs) and organic fluorescent dyes covalently linked by a peptide sequence specific to the protease of interest. In the absence of proteolytic activity (Figure 4e), FRET occurs between the QD and the organic dye, resulting in a yellow/orange emission signal. If the peptide is cleaved due to the proteolytic activity of the protease of interest, the dye diffuses away from the QD, leading to a green emission from the QDs. To bring this sensing method suitable to the PON, the authors demonstrated that a battery-powered LED as the excitation source and a smart phone can be used to quantitatively determine protease activities within 5 min.

Krull and co-workers developed different sensing systems deployed at PON, using a paper-based design. The biosensors are based on the transduction of nucleic acid hybridization [84,85,87,91,92] and both a direct and a sandwich format assay were developed, both based on paper-immobilized quantum dot-oligonucleotide conjugates (Figure 4f, sandwich assay format shown) [92,93]. Hybridization events in both formats brings the QD into close proximity with Cy3 [92]. Although the technology was first developed using lab-based epifluorescence, the researchers then moved to more PON-friendly equipment. They reported the adaptation of the test to paper substrate, combined with a handheld UV lamp as the excitation source and an iPad camera for the ratiometric fluorescence emission analyses. In comparison with the use of sophisticated laboratory equipment—in this case, an epifluorescence microscope—the authors reported a sensitivity approximately one order of magnitude less sensitive when using the portable equipment. The PON test described above was also used as the basis of rapid diagnostic tests, to detect a single nucleotide polymorphism indicative of spinal muscular atrophy [87] and a three-base replacement used in the diagnosis of cystic fibrosis [86].

Krull and co-workers also used a similar oligonucleotide-based system where a quantum dot was conjugated to a single-stranded DNA aptamer that specifically binds the cancer biomarker protein epithelial cell adhesion molecule (EpCAM), immobilized on paper [84]. A conjugate of Cy3 with an oligonucleotide complementary to the aptamer is added to the immobilized construct. FRET between the QD and an added Cy3-DNA conjugate could be observed in the absence of EpCAM. When EpCAM is present, it displaces the Cy3-DNA conjugate from the aptamer, resulting in a loss of FRET signal (Figure 4g). Multiple biosensors can be delivered in this way at the PON, including smaller aptasensors, which have been used to detect antibiotics in milk [88]; however, these approaches have not yet been adopted commercially.

#### 4.2.3. FRET Incorporating Upconversion Nanoparticles (UCNPs)

Luminescence resonance energy transfer (LRET) is a type of RET between upconverting nanoparticles (UCNPs), such as lanthanides, and a FRET acceptor. Translation of LRET biosensors for PON applications was also achieved by using PADs by Krull and co-workers [94]. The authors used UCNPs as the FRET donor and QDs as the acceptors to build a multiplexed paper-based assay for the simultaneous detection of three diagnostic markers for the bacterium *E.coli* [95]. Although multiplexing of the biosensor devices was achieved on paper, analyses of the paper strips were carried out by using an epi-fluorescence microscope, which is incompatible with PON applications. This highlights the limitations sometimes encountered with FRET-dependent systems that have only been demonstrated by using bulky, sophisticated laboratory equipment.

In another example of UCNP-based sensing, Wang and co-workers successfully adapted a nanosensor for PON detection of an organophosphonate nerve agent (Figure 4h) [89]. The biosensor development was initially carried out by using sophisticated laboratory equipment, after which the authors also reported that the exposure of the paper-strip biosensor to the nerve agent of interest leads to the emission of blue light that is visible with the naked eye.

#### 4.2.4. FRET-Based Systems Using Time Resolved Measurements

Time-resolved FRET (TR-FRET) involves measuring the fluorescence lifetimes of the FRET components instead of taking ratiometric measurements between donor and acceptor signal. Traditionally, TR-FRET has required heavy and complex lab-based machines, such as plate readers. For instance, commercially available assays, such as BRAHM’s TRACE^®^ assays, are applied on the KRYPTOR device [96], which is a 54 kg benchtop device, which is less suitable for on-site testing. Recently however, ProciseDx has produced a more PON-friendly device [97]. The shoe-box-sized device will allow the analysis of complex fluid samples, such as finger prick whole blood that can be inserted into the device via cartridges that contain the biosensor reagents, followed by their automatic analysis. Another user-friendly feature lies within the automatic mixing of sample and biosensor in the cartridge, removing a source of error through pipetting. No assays for the ProciseDx are commercially available at this point, but it has been announced that the first assay will be a 5 min test to quantitate the inflammatory marker C-reactive protein [98].

**Figure 5:**
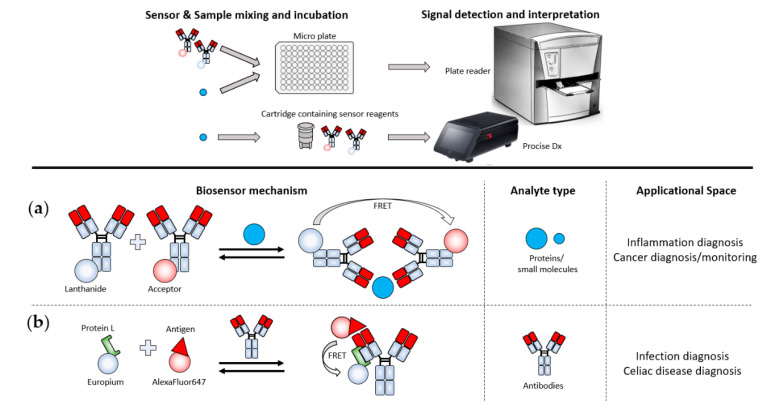
Examples of Lanthanide-FRET (LRET) applications that are potentially suitable for on-site testing. (**a**) Commonly applied TR-FRET technologies rely on a sandwich-based homogeneous assay, where two antibodies targeting different epitopes of the analyte are labeled with a lanthanide energy donor, while the other is labeled, with an organic dye or fluorescent protein, as the energy acceptor. Binding of both antibodies enables FRET between the lanthanide and acceptor which is measured by their altered fluorescence lifetimes. (**b**) Protein L, an antibody light-chain-binding protein [102], is labeled with Europium. Antigens to an antibody of interest are labeled with the organic dye AlexaFluor647 (LFRET) [103]. If a sample contains antibodies against the antigen–dye fusion, FRET occurs between Protein-L-Europium bound to the light chain of the antibody and the Antigen–AlexaFluor647 fusion. Image of the ProciseDx device was used with permission from ProciseDx.

The translation of TR-FRET from bulky bench-top devices into reliable portable detection devices would open up a myriad of options for on-site applications, as a wide range of TR-FRET assays is already commercially available [96,99] A range of other TR-FRET assays have also been described, including for the diagnosis of sepsis, cancer, disorders, virus infections [100] or celiac disease [101] (Figure 5).

## 5. Conclusions and Perspectives for RET-Based PON Systems

In recent years, we have seen tremendous progress in translating a range of laboratory-based RET biosensors into detection methods suitable for point-of-need testing. Improvements have occurred in multiple areas. These involve improved optical donors and acceptors, expanding the range of molecular architectures so as to creatively link an ever-expanding variety of biological recognition elements, developing improved assay matrices and miniaturizing suitable detection devices. While it is unlikely to see handheld all-in-one detection tools, such as the glucose meter, any time soon, due to their necessity of at least a photon-collecting component, RET-assays are quantitative and can be highly sensitive. This opens up their application to areas where trace-level analyses, such as for protease activity or antibiotics, and/or where quantitative measurements are needed rather than simply flagging the presence of an analyte at some (difficult to standardize) threshold. In the clinical area, such requirements often occur in the case of measuring inflammatory or metabolic markers.

A majority of RET-based techniques that can be applied at the point-of-need are amplification-free, allowing tests that deliver results within a few minutes. Gold standards such as PCRs for the detection of miRNAs or gene fragments, HPLC for the analysis of small molecules and ELISAs for the quantitation of proteins and small molecules are still too time-consuming, are user unfriendly and require specialized instruments to be deployed at the point-of-need, despite ongoing attempts to mitigate these disadvantages. On the other hand, RET-techniques hold the potential to deliver a step-change improvement in the speed and simplicity of point-of-need testing. Ultimately, the use-case is what determines the assay-time requirements. For example, clearing a food-processing line of allergens before commencing with a different product demands rapid, sensitive and quantitative results. Similarly, certain medical conditions, such as drug intoxication or infection, may benefit from test results that can be delivered within minutes.

Although no RET-based point-of-need applications are fully commercial yet, we see a clear drive towards developing FRET methods to meet the requirements at the PON. However, the inherent requirement of FRET for bulky illumination sources, such as fluorescence microscopes [52,54], lasers [53,104], arc lamps [85] or LEDs [83], is hindering the development of miniaturized or portable devices. In addition, FRET-based techniques suffer from high background levels in most complex samples, especially those containing autofluorescent molecules. The use of infrared-shifted FRET systems, enabling excitation wavelengths that do not trigger autofluorescent molecules, or applying TR-FRET can circumvent issues stemming from high background levels. Especially, TR-FRET is a rapid and sensitive alternative to sensitized FRET techniques, which could be a game changer at the point-of-need when smaller detection devices, such as the ProciseDx, with an easy-to-handle cartridge system, become widely available.

Striking progress has been seen in the field of RET biosensors that do not require an external light source, especially for biosensors incorporating BRET as the transduction modality. The Cybertongue^®^ technology is approaching PON acceptance through a solid state BRET detection system within a microfluidics- and temperature-controlled reaction environment [48]. This system can be applied at the point-of-need, for instance in food production facilities, with minimum sample preparation and limited need for operator training while maintaining highly reproducible results. Another approach focuses on truly portable sensing systems that are applied on PADs and analyzed by using a smart phone [73,74,81]. Such tools lend themselves to applications in resource-limited or highly time critical settings, such as for certain medical conditions; however, it remains to be seen if they can meet regulatory standards for sensitivity and reliability in clinical use. We expect their developers to focus strongly on demonstrating these features in the short-to-medium term.

With all of these development pathways occurring in parallel, we hope to see further rapid advances in the field, particularly as the advantages of different systems are combined and adapted. Moreover, we are reasonably optimistic that the next five years will see some of the technologies we have briefly reviewed herein taking their place in the clinic and the factory, alongside well-established LF and EC platforms.

## Data Availability

Not applicable.
